# Social disadvantage is associated with impaired increase in salivary diurnal melatonin amplitude throughout pregnancy

**DOI:** 10.1093/sleepadvances/zpaf064

**Published:** 2025-09-30

**Authors:** Ronald T McCarthy, Erin L Reinl, Patricia K Strutz, Peinan Zhao, Andrea Esparza, Emma R Sass, Meridith Schoening, Pranita Kaginele, Naiqi Shi, Madeline Canal, Jessica E Chubiz, Justin C Fay, Emily S Jungheim, Nandini Raghuraman, Lindsey N Kent, Rebecca C Cox, Antonina I Frolova, Erik D Herzog, Sarah K England

**Affiliations:** Department of Obstetrics and Gynecology, Washington University School of Medicine, St. Louis, MO, United States; Center for Reproductive Health Sciences, Washington University School of Medicine, St. Louis, MO, United States; Department of Obstetrics and Gynecology, Washington University School of Medicine, St. Louis, MO, United States; Center for Reproductive Health Sciences, Washington University School of Medicine, St. Louis, MO, United States; Department of Anesthesia, Washington University School of Medicine, St. Louis, MO, United States; Department of Obstetrics and Gynecology, Washington University School of Medicine, St. Louis, MO, United States; Department of Obstetrics and Gynecology, Washington University School of Medicine, St. Louis, MO, United States; Department of Obstetrics and Gynecology, Washington University School of Medicine, St. Louis, MO, United States; Department of Obstetrics and Gynecology, Washington University School of Medicine, St. Louis, MO, United States; Department of Obstetrics and Gynecology, Washington University School of Medicine, St. Louis, MO, United States; Department of Obstetrics and Gynecology, Washington University School of Medicine, St. Louis, MO, United States; Department of Obstetrics and Gynecology, Washington University School of Medicine, St. Louis, MO, United States; Department of Obstetrics and Gynecology, Washington University School of Medicine, St. Louis, MO, United States; Department of Biology, University of Rochester, Rochester, NY, United States; Department of Obstetrics and Gynecology, Washington University School of Medicine, St. Louis, MO, United States; Department of Obstetrics and Gynecology, Northwestern University Feinberg School of Medicine, Chicago, IL, United States; Department of Obstetrics and Gynecology, Washington University School of Medicine, St. Louis, MO, United States; Department of Obstetrics and Gynecology, Washington University School of Medicine, St. Louis, MO, United States; Center for Reproductive Health Sciences, Washington University School of Medicine, St. Louis, MO, United States; Department of Psychological and Brain Sciences, Washington University in St. Louis, St. Louis, MO, United States; Department of Obstetrics and Gynecology, Washington University School of Medicine, St. Louis, MO, United States; Center for Reproductive Health Sciences, Washington University School of Medicine, St. Louis, MO, United States; Department of Biology, Washington University in St. Louis, St. Louis, MO, United States; Department of Obstetrics and Gynecology, Washington University School of Medicine, St. Louis, MO, United States; Center for Reproductive Health Sciences, Washington University School of Medicine, St. Louis, MO, United States

**Keywords:** melatonin, pregnancy, social adversity, women’s health

## Abstract

**Study Objectives:**

Melatonin regulates daily rhythms and is important for maintaining a healthy pregnancy. Certain socioeconomic factors may affect melatonin release. This study evaluates whether the increase in melatonin with advancing gestation is associated with social disadvantage.

**Methods:**

Data were prospectively collected from a socioeconomically diverse cohort of participants with singleton pregnancies (*n* = 921) at a Midwest academic center. Participants self-collected saliva every four hours over a 24-hour period once per trimester. Diurnal melatonin concentration was measured, and for each trimester, the maximum and mean diurnal melatonin concentration values were obtained. Cosinor-fitting was performed to obtain peak, mesor, and amplitude values, and melatonin profiles were also analyzed by calculating area under the curve. Participants were dichotomized by high and low social disadvantage score (SDS), and diurnal melatonin parameters were compared between participants with high and low SDS.

**Results:**

Mean diurnal melatonin concentration increased at an average rate of 0.19 pg/mL/week, and amplitude increased by 0.04 pg/mL/week. Participants with high SDS had significantly lower diurnal melatonin concentration amplitudes, means, mesors, and peaks than those with low SDS. Participants with high SDS had a 2.19 [95%CI = 1.94, 2.47] adjusted relative risk for low diurnal amplitude melatonin and had a smaller increase in diurnal melatonin amplitude over weeks of pregnancy than those with low SDS (−0.04 vs. 0.11 pg/mL/week, *p*<.001).

**Conclusions:**

Average salivary diurnal melatonin concentration increases across pregnancy, but the degree of increase varies among pregnant participants and is associated with social disadvantage.

**Statement of Significance**

During pregnancy, melatonin increases with gestational age and may promote placental homeostasis, fetal maturation, and uterine contractions. In this study, we evaluated whether the increase in melatonin with advancing gestation is modified by social disadvantage. We found that salivary diurnal melatonin concentration mean and amplitude increased as pregnancy progressed, but this increase was blunted or even reversed in participants with high social disadvantage scores. Future research should evaluate strategies to intervene, either via behavioral changes or pharmacologic therapy, to mitigate the negative impacts of social disadvantage on maternal melatonin rhythms.

## Introduction

During pregnancy, the body adapts to support the developing fetus, attenuate stressors, and regulate parturition timing. These adaptations include increasing concentrations of the hormones cortisol, estrogen, and progesterone and reducing total daily activity [1–6]. Additionally, pregnant individuals have longer sleep duration than before pregnancy and shift their sleep period onset earlier during early gestation [7, 8]. An important hormone in sleep regulation is melatonin, which undergoes daily cyclic variation, rising in the evening, peaking in the early morning, and remaining low during the day. Melatonin promotes sleep, entrains physiological processes with daily and seasonal rhythms [9], has antioxidant effects [10], and appears to have important roles in human reproduction [11–14]. During pregnancy, melatonin increases with gestational age and has been suggested to promote placental homeostasis, fetal maturation, and uterine contractions [11–16]. Along with higher concentrations of melatonin during the third trimester of pregnancy, Sharkey et al. found elevated melatonin receptor type 2 in human term laboring myometrium [17].

Melatonin is produced by the pineal gland, regulated by the light/dark cycle of the 24-hour day, and synthesized primarily in the dark phase. The precursor L-tryptophan is first converted to 5-hydroxytryptophan by tryptophan hydroxylase, which is highly expressed during the dark phase. The 5-hydrooxytryptophan is then converted to serotonin by aromatic amino decarboxylase. At night, the neurotransmitter norepinephrine activates the pineal gland β-adrenergic receptors. This increases cellular cAMP and expression of serotonin N-acetyl transferase, which converts serotonin to N-acetyl -serotonin. N-acetyl-serotonin is then methylated by hydroxyindole-o-methyl transferase to become melatonin, which is secreted into circulation.

The primary role of melatonin is to regulate daily rhythms of physiological functions, such as sleep duration, temperature, and blood pressure [12]. Melatonin also contributes to regulation of weight and mammalian reproduction. Disruption in sleep and exposure to artificial light at night affect the timing of production and secretion of melatonin by the pineal gland [18]. Thus, melatonin is considered the gold standard biological marker for assessing the central circadian clock [12, 18].

Much of our understanding of changes in melatonin concentration in pregnancy has come from animal models and small human cohort studies. In a Japanese cohort comparing 7 non-pregnant participants to 30 participants with singleton pregnancies, Nakamura et al. collected blood samples twice per 24-hour period: at 0200 (nighttime) and 1400 (daytime) at varying gestational ages. They found that melatonin peaked in the third trimester and dropped to non-pregnant concentrations immediately after delivery [13]. Ejaz et al. collected blood samples in the morning from a British cohort of 26 pregnant women. They found that melatonin concentration increased significantly during pregnancy, reached the peak in the third trimester, and decreased abruptly after delivery [11]. This group further showed that the placenta is a major contributor of maternal melatonin during the later stages of pregnancy. These studies, although informative, are limited by the small sizes of the cohorts and the racial homogeneity or lack of reported racial, ethnic, and socioeconomic composition of the cohorts.

This lack of diversity in human studies of melatonin in pregnancy is important because people with high social disadvantage are more likely exposed to factors that alter proper melatonin regulation than those with low social disadvantage. Many studies have indicated that those who are of low socioeconomic status have more sleep disturbances [18–25] and are exposed more to light at night [26]. These factors, in addition to shift work [27], can negatively affect physiology and behavior [28, 29] and may affect pregnancy. For example, shift work and sleep deprivation have been linked to miscarriage, low birth weight, and preterm birth [30–36]. Moreover, low melatonin concentrations are associated with preeclampsia in human parturients and abnormal parturition timing in animal models [12, 37].

A common strategy to measure melatonin as a biomarker of circadian rhythms is to perform controlled in-laboratory studies. However, these methods have limited ability to provide information about real-world disruption of the normal circadian rhythm (chronodisruption), particularly when the context of interest (e.g. social disadvantage) is difficult to replicate in the laboratory. In such cases, field studies of diurnal melatonin (i.e. 24-hr oscillations in melatonin arising from a combination of the endogenous circadian rhythm and external influences) may provide more ecologically valid information to understand the impact of context on daily rhythms in physiology. For example, Jensen et al. measured diurnal melatonin during shift work to examine adaptation to night shifts and whether the degree of adaptation was influenced by the number of consecutive shifts [38]. Likewise, studies of patients in intensive care units revealed that diurnal melatonin output was lower and later than in reference patients [39, 40] and that lower diurnal melatonin amplitude was associated with worse clinical outcomes [41]. These studies highlight the utility of assessing diurnal melatonin as a measure of chronodisruption in a complex, real-world context.

Individuals with high social disadvantage are at greater risk of adverse reproductive outcomes than those with low social disadvantage. Although many of the social factors that negatively affect sleep are more common in people with high social disadvantage than in those with low social disadvantage [19–21, 26, 42, 43], the relationship between social disadvantage and melatonin has not been defined in pregnancy. Here, our objectives were to define the maternal 24-hour melatonin profile changes throughout pregnancy in a large, diverse cohort and examine the extent to which these changes associate with social disadvantage.

## Methods

### Study design and participants

This was a secondary analysis of samples and data from a prospective cohort study addressing possible causes of preterm birth [44]. Participants were included in this analysis if they were English speaking, were between ages 18 and 45 years, had a singleton pregnancy that resulted in a live birth, and provided an adequate volume of saliva from at least five times of day during at least one trimester. Potential participants were excluded if they had a fetal anomaly that affects the timing of birth or conceived via in vitro fertilization. Participants were recruited at their initial appointment in one of two obstetric clinics between January 30, 2017, and November 27, 2019 [44]. The study was approved by the Washington University in St. Louis Institutional Review Board, and written informed consent was obtained from all participants. Trained obstetric research nurses collected baseline demographics and antenatal characteristics from electronic medical records. Participants’ income-to-needs ratios (I/N) were derived by dividing their annual income at enrollment by their family size-adjusted need as determined by the United States Census Bureau money-income poverty thresholds [45].

### Saliva collection

**Figure 1:**
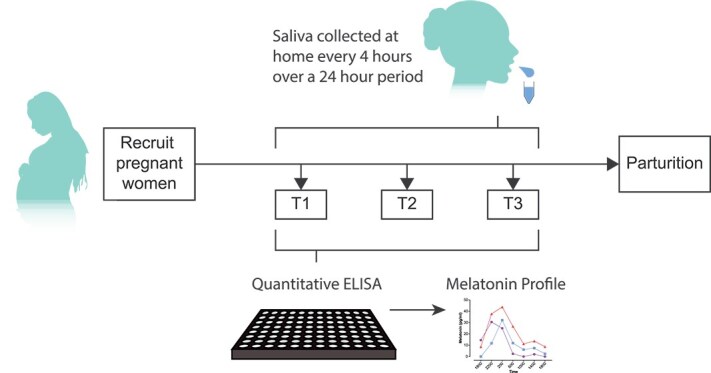
Schematic of the study design. Participants were asked to collect saliva at 4-hour intervals over a 24-hour period (up to 7 samples) outside the clinic once each trimester. An enzyme-linked immunosorbent assay was used to measure salivary diurnal melatonin concentration, and values were plotted against the times they were collected.

We analyzed salivary diurnal melatonin because saliva can be collected easily and non-invasively. Salivary melatonin concentration correlates with serum melatonin concentration [46, 47], has been used in multiple studies [48–51], including those assessing diurnal melatonin in complex populations (e.g. [38, 40]), and is the gold standard biochemical marker for determining dim light melatonin onset [52, 53]. All participants were asked to provide saliva samples once each trimester (first trimester: 7 to 13 weeks’ gestation, second trimester: 14 to 27 weeks, and third trimester: ≥ 28 weeks) (Figure 1). Some participants who were recruited at the end of the first or beginning of their second trimester were asked to provide saliva samples both early and late during the second trimester.

We instructed participants to provide saliva samples starting at 1800, then every 4 hours for 24 hours (at least 6 samples), thus ideally collecting a sample around 0200 when serum melatonin concentration typically peaks. We did not require participants to collect saliva samples while seated in dim light, as is typical in many studies, because we aimed to measure melatonin concentrations in real-world conditions. Therefore, participants collected samples in their usual environments and lighting conditions. Each trimester, participants were provided with seven Salivette tubes (Sarstedt SAR-511534500) and instructions on how to collect saliva. Each Salivette tube consisted of a 15 mL conical tube containing a synthetic roll-shaped swab in a suspended insert. Instructions were to (1) collect a clear sample of saliva with no contamination of food, lipstick, blood, or any other extraneous materials at least 30 minutes after a meal, oral intake of pharmaceutical drugs, or teeth cleaning; (2) remove the synthetic swab from the collection tube without it touching their hands, insert it between cheek and gum, gently chew for 1-2 minutes or until the mouth salivated, and then use their teeth to return the swab into the suspended insert within the collection tube; and (3) immediately record the date and actual time of collection on the tube, seal the tube with the blue cap, and store the tube in a standard home freezer until it could be delivered to the clinical staff. When tubes arrived in the clinic, they were thawed and centrifuged for 2 minutes at 1000 x *g*. Each saliva sample was approximately 1 mL. The saliva was aliquoted into cryovials (Corning 2.0 mL external thread #430659) and stored at −80°C until ready for analysis.

### Diurnal melatonin measurements

Frozen aliquoted saliva samples were thawed, centrifuged at 1500 x *g* for 15 minutes, and analyzed with a Saliva Melatonin ELISA kit (Salimetrics 31-3402-5) according to the manufacturer’s instructions. Samples were measured on a BioTek Eon plate reader that uses Gen5 software (version 2.07). Absorbance was measured at 450 nm, and a secondary filter correction was performed at 620 nm. All samples were measured in duplicate. Sample concentrations were calculated according to a standard curve with melatonin concentrations between 0.78 and 50 pg/mL. The assay has a threshold sensitivity of 1.37 pg/mL and a coefficient of variation of 23.6%, yielding a functional sensitivity of 1.42 pg/mL. Any samples with melatonin concentrations above 50 pg/mL were diluted and re-assayed until values fit within the standard curve. The corrected melatonin concentration values were then calculated according to the dilution factor. The intra- and inter-assay coefficients of variation during the study were 5.4% and 8.9%, respectively.

### Social disadvantage score

The Social Disadvantage Score (SDS) was derived to capture a latent construct of maternal socioeconomic adversity by using Confirmatory Factor Analysis within a Structural Equation Modeling framework [54, 55] This approach was chosen to account for the high correlations among various dimensions of adversity and to distinguish their unique contributions to maternal and fetal outcomes.

To estimate SDS, we included a set of observed socioeconomic and environmental indicators collected throughout pregnancy. These variables included: Household I/N Ratio; Area Deprivation Index percentile, based on national census block rankings; Healthy Eating Index derived from the National Institutes of Health Diet History Questionnaire; Highest level of educational attainment; and Health insurance status.

These observed variables were used to estimate an individual-level latent SDS, representing cumulative social disadvantage. A score of >0 was interpreted as higher disadvantage, based on the centered factor distribution. Race was not included in the SDS model because of its high correlation with other disadvantage indices; model testing confirmed that including race did not improve explanatory power once variables such as area deprivation index and discrimination exposure were accounted for.

### Statistical analyses

**Figure 2:**
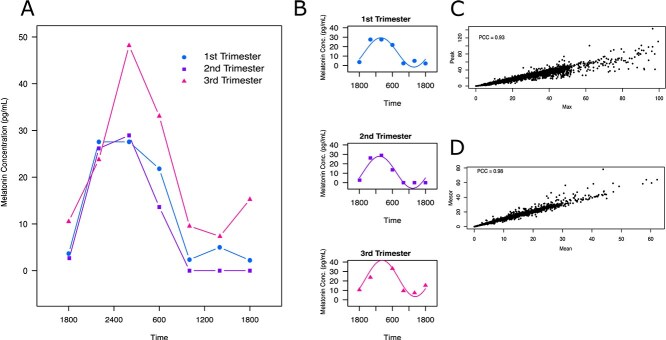
Raw and cosinor-fitted melatonin profiles. (A) Diurnal melatonin profiles from a representative pregnant woman collected over each trimester. (B) The corresponding Cosinor-fitted data, with the maximum, defined as the highest raw melatonin concentration, peak as the maximum of the Cosinor fit, Mesor as the mean of the Cosinor, and amplitude as the difference between the peak and the Mesor. Correlations between (C) peak and maximum values, and (D) mean and mesor values. PCC = Pearson’s correlation coefficient.

Participants were included in the study if they provided at least five saliva samples within a 24-hour period. To ensure accurate estimation of diurnal melatonin rhythms, only participants who provided a sample at 0200 (2:00 a.m.) (scheduled time)—the expected peak of melatonin secretion—were included in the analysis (921/938 study participants). Diurnal melatonin concentration rhythms were analyzed by fitting melatonin concentrations against the actual collection time of day recorded on the tubes by the participants. Melatonin maxima and means were obtained from the raw data (Figure 2, A). To better model the rhythmic secretion pattern of melatonin and reduce variability from discrete sampling times, we applied cosinor fitting by using the least squares method to fit a sine function with a period of 24 hours to the collected melatonin concentration time series (cosinor R package) [56]. From the fitted model, we extracted the mesor (fitted mean), peak (fitted crest), and amplitude (mesor to peak) (Figure 2,B). The fitted peaks highly correlated with the absolute maxima (Figure 2,C), and the means highly correlated with the mesors (Figure 2,D), indicating high robustness of the cosinor fitting method. A linear mixed effect model (lme4 R package) was used to consider both the populational average melatonin increase across pregnancy and patient-specific variation. A linear mixed effect model allows the use of gestational age as a continuous variable and estimates the rate of melatonin change across pregnancy. Gestational age (in weeks) was modeled as a fixed longitudinal effect to estimate the overall slope of change in melatonin measures (mean, amplitude, mesor) across pregnancy. Participant ID was included as a random effect to account for within-subject correlation due to repeated measures.

To quantify overall melatonin exposure across a 24-hour cycle, we calculated the area under the curve (AUC) by using salivary melatonin concentration measurements and exact sample collection times. To address irregular sampling times and occasional missing values, we first applied linear interpolation to estimate melatonin concentrations at the standard time points based on the available data. AUC was computed by using the trapezoidal rule, which approximates the area under the concentration-time curve by summing the areas of trapezoids formed between each pair of adjacent time points. This method accounts for variations in sampling intervals and provides a robust estimate of total melatonin secretion over the 24-hour period.

In analyses to assess differences in melatonin concentration across pregnancy, participants were dichotomized according to high or low mean melatonin concentration amplitude, which was calculated by taking the average diurnal melatonin amplitude for all time points in pregnancy at which they provided usable samples. In other analyses, participants were dichotomized by high (>0) or low SDS (Supplemental Figure 1).

Baseline demographics and melatonin characteristics were compared between participants with high and low SDS by using *X*^2^ for categorical variables and the Welch student’s two-sample *t*-test or Wilcoxon rank-sum test for continuous variables, as appropriate. Normality was tested with the Shapiro-Francia test. Median and inter-quartile range values were reported for the melatonin characteristics among the patient cohort who provided samples at each trimester. The melatonin parameters were stratified by SDS < 0 and compared with the Wilcoxon rank-sum test. The melatonin parameter change rate across pregnancy was stratified by SDS < 0 and tested by linear mixed effect model. The *p*-value of interaction between SDS and melatonin parameter change rate was calculated with Wald’s t-test. Multivariable logistic regression was used to adjust for potential confounders, which were selected based on results of univariate analysis (Table 1) and biologic plausibility (maternal age, body mass index, cigarette use, pre-existing hypertension, and pregestational diabetes) in evaluating the relationship between SDS and melatonin amplitude. All statistical analyses were conducted with R (version 4.3).

**Table 1:** Demographics of study cohort

	**Total Cohort** **(*n* = 921)**	**Low SDS (< 0)** **(*n* = 461)**	**High SDS (> 0)** **(*n* = 460)**	**Test statistics** **(degrees of freedom)**	***p* Value**
Age (years) at enrollment	28.25 ± 5.43	30.62 ± 4.48	25.88 ± 5.27	t(895) = 14.7	< 0.001
BMI (kg/m^2^) at first visit	29.06 ± 8.24)	27.30 ± 7.15	30.83 ± 8.87	t(878) = -6.7	< 0.001
Obese (%, BMI > 30 kg/m^2^)	340 (36.9)	119 (25.8)	221 (48.0)	χ^2^(1) = 47.9	< 0.001
Smoker (%)	93 (10.1)	10 (2.2)	83 (18.1)	χ^2^(1) = 61.9	< 0.001
Nulliparous (%)	379 (41.2)	246 (53.4)	133 (28.9)	χ^2^(1) = 55.8	< 0.001
Race (%)				χ^2^(4) = 449.4	< 0.001
Black or African American	469 (50.9)	75 (16.3)	394 (85.7)		
White	412 (44.7)	353 (76.6)	59 (12.8)		
Asian	18 (2.0)	18 (3.9)	0 (0.0)		
American Indian or Alaskan Native	3 (0.3)	3 (0.7)	0 (0.0)		
Unknown or Other	19 (2.0)	10 (2.7)	7 (1.5)		
Employment (%)				χ^2^(3) = 106.5	< 0.001
Yes	667 (72.4)	402 (87.2)	265 (57.6)		
No	185 (20.1)	36 (.8)	149 (32.4)		
Student	28 (3.0)	12 (2.6)	16 (3.5)		
Unknown	41 (4.5)	11 (2.4)	30 (6.5)		
INR Range (%)				χ^2^(3) = 719.6	<0.001
Below poverty (<1.0)	219 (24.3)	0 (0.0)	219 (48.9)		
Near poverty (1.0-1.5)	128 (14.2)	5 (1.1)	123 (27.5)		
Moderate (1.5-3.0)	150 (16.6)	51 (11.3)	99 (22.1)		
High (>3.0)	404 (44.8)	397 (87.6)	7 (1.6)		
Hypertension (%)	96 (10.4)	26 (5.7)	70 (15.2)	χ^2^(1) = 21.4	< 0.001
Pregestational Diabetes (%)	23 (2.5)	9 (2.0)	14 (3.1)	χ^2^(1) = 0.72	0.395

Values are mean ± SD or N (%).

SDS, social disadvantage score; BMI, body mass index.

Continuous variables are presented as mean ± SD and compared using Student’s *t*-test. Categorical variables are presented as n (%) and compared using χ^2^ test.

## Results

### Participant characteristics

**Figure 3:**
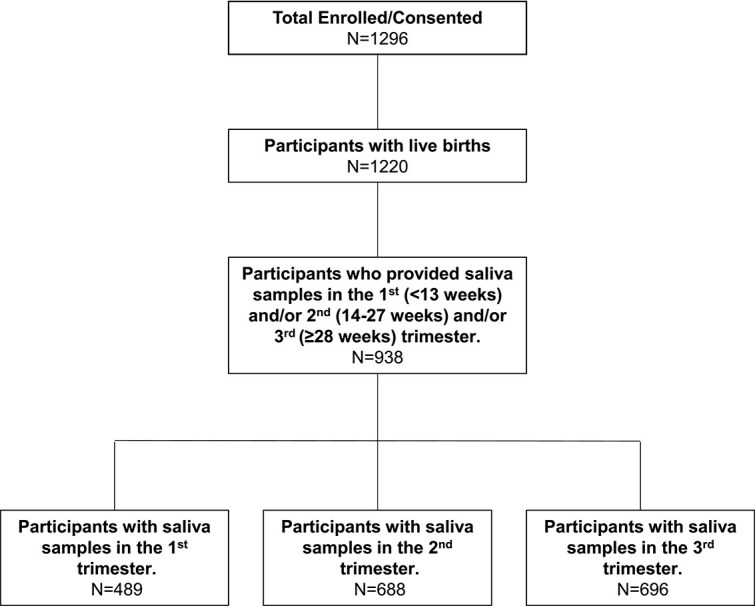
Participant enrollment flowchart.

Of 1296 pregnant patients enrolled in the parent study, 1220 had live births, and of these, 938 provided saliva samples for analysis. Of the 938 participants, 489 had usable first trimester measurements, 688 had usable second trimester measurements, and 696 had usable third trimester measurements (Figure 3). Additionally, 286 participants had usable measurements from all three trimesters (Supplemental Table 1). In brief, the average age was 28 years (range 18 to 42). Over one-third (36.9%) of participants had obesity, which is near the national average of 39.7% for women ages 20–39 years [57]. The cohort included 50.9% Black participants. Approximately one-quarter (23.1%) of participants were unemployed or students, and 38.5% were living below (I/N ≤ 1) or near (I/N = 1-1.5) the poverty threshold at enrollment. Participants with high SDS (>0) were, on average, 5 years younger and more likely to have obesity, to be multiparous, to have pre-existing hypertension, and to smoke cigarettes than participants with low SDS (Table 1).

### Timing of melatonin sample collection

Participants provided saliva samples at 10.7 ± 2, 21.2 ± 3.6, and 33.4 ± 2.8 weeks for trimesters 1, 2, and 3, respectively. The median timing of saliva collection was 0 minutes from the requested time, with 75% of saliva collections occurring within 5 minutes of the requested time (Supplemental Table 2). In all trimesters, ~90% of participants provided samples within 30 minutes of the requested time (Supplemental Table 3).

### Maternal salivary diurnal melatonin concentrations during pregnancy

We examined diurnal melatonin concentration amplitude, mesor, mean, maximum, and AUC for each trimester of pregnancy (Table 2). We applied a linear mixed effect model to determine the rate of change for diurnal melatonin over the course of pregnancy. All diurnal melatonin parameters significantly increased as pregnancy progressed (Supplemental Figure 2, Table 3). In a sub-analysis of participants who provided samples in every trimester (*n* = 286), the diurnal melatonin amplitude increased 0.06 ± 0.02 pg/mL per week (Supplemental Table 4), which was similar to the slope of melatonin amplitude across pregnancy in all participants (Table 3). AUC analysis (Table 4) also showed that melatonin concentration increased over the three trimesters.

**Table 2:** Comparison of diurnal melatonin profile parameters across trimesters

Trimester	N	Amplitude (pg/mL) (Median, IQR)	Mesor (pg/mL) (Median, IQR)	Mean (pg/mL) (Median, IQR)	Maximum (pg/mL) (Median, IQR)	AUC (pg/mL) (Median, IQR)
1	496	9.5 (4.9, 14.9)	11.9 (7.4, 16.4)	11.3 (7.1, 15.5)	26.5 (16.0, 37.4)	292.6 (181.7, 405.4)
2	722	9.1 (3.9, 14.8)	13.2 (8.3, 18.4)	12.6 (8.0, 17.7)	27.2 (17.1, 40.4)	320.5 (200.9, 457.3)
3	735	9.2 (3.0, 16.9)	15.9 (10.0, 22.1)	15.0 (9.9, 21.1)	31.7 (18.1, 44.4)	385.0 (238.3, 531.5)

Values are estimated coefficients from linear mixed-effects models, shown as median (Q1, Q3)

**Table 3:** Rate of change for diurnal melatonin profile parameters according to the linear mixed model

Melatonin parameter (Mean)	Baseline (pg/mL)	Slope by Week (pg/mL per week)	*p* value
Amplitude	9.7 ± 0.4	0.04 ± 0.02	0.03
Mesor	10.8 ± 0.2	0.19 ± 0.02	< 0.001
Mean	9.9 ± 0.4	0.17 ± 0.02	< 0.001
Maximum	25.6 ± 0.8	0.16 ± 0.03	< 0.001
AUC	267.0 ± 11.8	4.5 ± 0.5	<0.001

Values are estimated coefficients from linear mixed-effects models. *p* value tests significant slope.

**Table 4:** Association between SDS and diurnal melatonin profile parameters by trimester

Melatonin parameters	Low SDS (< 0)	High SDS (>0)	*p* Value
**Trimester 1**			
Amplitude (pg/mL)	11.6 (6.9, 17.0)	5.7 (2.6, 11.3)	< 0.001
Mesor (pg/mL)	12.5 (8.6, 16.2)	10.1 (6.1, 15.8)	0.003
Maximum (pg/mL)	28.3 (19.4, 38.4)	20.4 (12.4, 31.9)	< 0.001
Mean (pg/mL)	11.9 (8.1, 15.5)	9.8 (6.0, 14.5)	0.004
AUC (pg/mL)	307.1 (207.5, 405.1)	244.6 (139.7, 393.2)	0.001
**Trimester 2**			
Amplitude (pg/mL)	11.8 (7.4, 17.3)	4.7 (2.4, 10)	< 0.001
Mesor (pg/mL)	14.3 (9.9, 18.8)	11.1 (7.0, 17.4)	< 0.001
Maximum (pg/mL)	31.3 (21.3, 41.5)	21.5 (12.1, 34.5)	< 0.001
Mean (pg/mL)	13.1 (9.2, 17.3)	10.7 (6.8, 16.6)	< 0.001
AUC (pg/mL)	349.0 (239.9, 464.1)	254.6 (167.6, 425.4)	<0.001
**Trimester 3**			
Amplitude (pg/mL)	14.4 (9, 19.2)	3.9 (1.9, 8.9)	< 0.001
Mesor (pg/mL)	17.4 (12.6, 22.4)	12.7 (7.6, 20.4)	< 0.001
Maximum (pg/mL)	36.4 (25.9, 45.6)	22.8 (13.0, 36.3)	< 0.001
Mean (pg/mL)	15.8 (11.9, 20.5)	12.0 (7.6, 19.6)	< 0.001
AUC (pg/mL)	426.2 (302.7, 537.3)	298.0 (177.5, 512.9)	<0.001

Values are median (95% confidence interval). SDS, social disadvantage score. Group comparisons were conducted by using Wilcoxon rank-sum test.

### Strong versus weak diurnal melatonin rhythms

**Figure 4:**
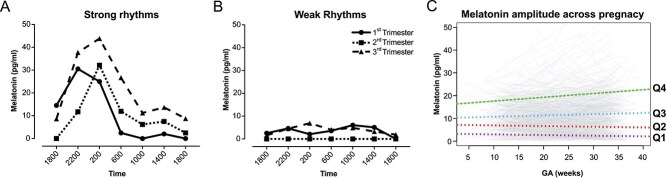
Participants with high melatonin amplitude in early pregnancy show increased amplitude by the end of pregnancy. (A, B) Example raw diurnal melatonin profiles showing (A) a participant with strong melatonin rhythms and (B) a participant with weak melatonin rhythms in all three trimesters. (C) Diurnal melatonin amplitude for all participants over the course of pregnancy. Dashed lines represent the generalized linear model for amplitude over the course of pregnancy after dividing participants into quartiles according to their mean diurnal melatonin amplitude over all timepoints.

While assessing normality of diurnal melatonin values, we noted two distinct melatonin profile types. Some participants had clear daily variation in melatonin concentrations that typically peaked around 0200 hours and reached the nadir between 1000 and 1800 hours. We refer to these as “strong” diurnal melatonin rhythms (Figure 4,A). Some participants had “weak” diurnal melatonin rhythms with lower peak diurnal melatonin concentrations (Figure 4,B). We hypothesized that participants with weak or strong rhythms differed in their diurnal melatonin trajectories during pregnancy. To test this, we subdivided participants into quartiles by the average melatonin amplitude across pregnancy (*n* = 230, 230, 231, and 230), with those in quartile 4 having the highest mean diurnal melatonin concentration amplitudes in all trimesters. We performed a linear mixed effect model analysis to fit the diurnal melatonin concentration as a function of gestational age and patient quartiles. In quartiles 3 and 4, but not in quartiles 1 and 2, diurnal melatonin amplitudes increased over the course of pregnancy (Figure 4,C). We then dichotomized the participants equally into high or low amplitudes according to the average amplitude for all timepoints at which usable samples were provided. The cutoff value was median 8.9 pg/mL. In those with high diurnal melatonin amplitudes, amplitudes increased at a rate of 0.12 pg/mL/week. In those with low diurnal melatonin amplitudes, amplitudes decreased at a rate of -0.04 pg/mL/week (*p*<.001 for interaction between participant group and slope).

### Association between SDS and diurnal melatonin amplitude

**Figure 5:**
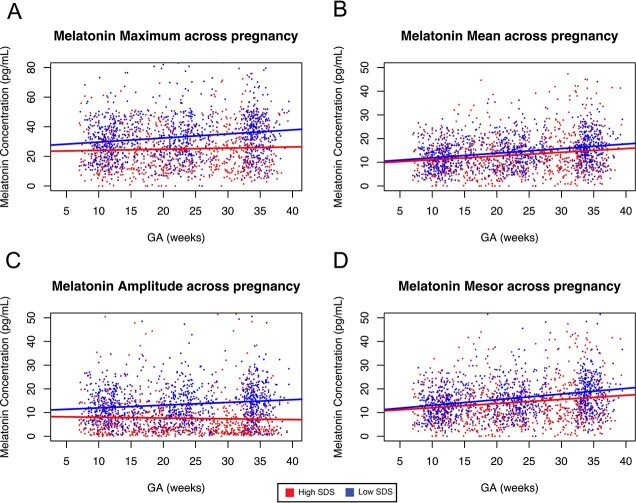
High SDS is associated with low diurnal melatonin maximum and amplitude. Daily melatonin (A) maximum, (B) mean, (C) amplitude, and (D) mesor plotted for all participants over the course of pregnancy. Dashed lines represent the generalized linear model for each statistic after dichotomizing individuals into high and low SDS groups.

Given the associations between socioeconomic status and sleep disruption [21, 22, 24, 58–60], we hypothesized that higher SDS would be associated with a smaller increase in diurnal melatonin amplitude across weeks of pregnancy. To test this, participants were dichotomized by SDS scores (> 0 and ≤ 0), and their diurnal melatonin profile parameters were compared at each trimester with the Wilcoxon rank-sum test (Table 4). All diurnal melatonin profile parameters throughout pregnancy were significantly lower in the high SDS group than in the low SDS group. The greatest difference was observed for amplitude in trimester 3 (3.7-fold difference between groups) (Table 4). In longitudinal analysis, melatonin maximum values for those in the high SDS group increased to a smaller degree than those for the low SDS group (Figure 5, A, Table 5). Furthermore, whereas amplitude increased for those in the low SDS group, it decreased for those in the high SDS group (Figure 5,C, Table 5). No group differences were observed for mean (Figure 5,B, Table 5).

**Table 5:** Association between SDS and diurnal melatonin change with gestation

	Low SDS (< 0)	High SDS (>0)	*p* value
Amplitude (pg/mL/wk)	0.11 ± 0.02	-0.04 ± 0.03	< 0.001
Mesor (pg/mL/wk)	0.23 ± 0.02	0.15 ± 0.04	0.03
Maximum (pg/mL/wk)	0.25 ± 0.04	0.06 ± 0.05	0.002
Mean (pg/mL/wk)	0.19 ± 0.02	0.15 ± 0.03	0.13
AUC (pg/mL/wk)	5.8 ± 0.6	3.1 ± 0.9	0.003

SDS, social disadvantage score. The linear mixed effect model was conducted, where Low SDS was considered as a fixed effect to the diurnal melatonin change across pregnancy. *p*-values were calculated by Wald t-test.

Lastly, we tested whether having a high SDS increased a participant’s risk for having a low diurnal melatonin amplitude, defined as below the median of 8.9 pg/mL, after adjusting for potential confounders. Participants with high SDS were more likely to have low amplitude than those with low SDS (aRR = 2.19, [95%CI = 1.94, 2.47]). The direction, magnitude, and significance were comparable between the full cohort and a sub-cohort only including those who provided samples within 30 minutes of the requested time windows (Supplemental Tables 5–7) and those with significant cosinor fits (Supplemental Table 8). This indicates that the effects observed in the cosinor analysis were not driven by timing nonadherence.

## Discussion

### Principal findings

In this large cohort of pregnant individuals, salivary diurnal melatonin concentration mean, amplitude, and AUC increased as pregnancy progressed, but this increase was blunted or even reversed in participants with high SDS. Our findings are consistent with those of previous studies suggesting that melatonin concentration increases across pregnancy. Notably, we overcame several limitations of previous studies. First, previous studies included small cohorts (*n* < 100), whereas we included samples from 921 participants. Second, in previous work, samples were collected at a single time of day and at only one or two timepoints during pregnancy [11, 13, 16]. In contrast, we measured diurnal melatonin at five or more timepoints over 24 hours in all three trimesters of pregnancy. Third, previous studies were often conducted with homogenous populations, whereas 49.9% of our participants had high social disadvantage and over 50% were Black.

The function of increasing melatonin across pregnancy is unknown but is likely relevant to healthy birth outcomes. Melatonin may protect the placenta and fetus from oxidative and nitrosative stress by scavenging free radicals and promoting antioxidant activity [10]. Maternal melatonin also helps synchronize the fetus to local light–dark cycles in rodents, suggesting a role in regulating fetal development [61]. Late in gestation, melatonin peak concentrations are thought to regulate uterine contractions. Specifically, uterine contractions are stronger at night when melatonin peaks, and night-time saliva melatonin concentration in the third trimester has been positively associated with contraction frequency [13, 62, 63]. Given these findings, an increase in daily mean melatonin secretion as pregnancy progresses may contribute to optimal reproductive outcomes.

Our large, diverse cohort allowed for in-depth real-world analysis of diurnal melatonin profiles throughout pregnancy. We found that, although average diurnal melatonin amplitude increased across pregnancy, the degree of increase varied between participants. Moreover, participants with high social disadvantage were 2.2 times more likely to have low diurnal melatonin amplitude than participants with low social disadvantage. The cause of lower melatonin in women with high social disadvantage is unknown. Stringhini et al. examined associations between socioeconomic status and several sleep outcomes and found that men and women with the lowest socioeconomic status had the poorest sleep outcomes. Moreover, women who held lower occupation positions had worse overall sleep disturbances [23]. Indeed, circadian disruption due to nightshift work results in decreased melatonin secretion, even under low-light conditions [64, 65]. This relationship is biologically plausible, as dampened melatonin concentrations have been attributed to disruptions in the sleep/wake cycle [66–68] and exposure to light at night [69–72], and individuals of low socioeconomic status and marginalized racial and ethnic identities [73] are exposed to more light at night [26]. Thus, habitual light exposure patterns, particularly excessive light at night, may contribute to the observed disruption in diurnal rhythms in women with high social disadvantage. Future research is needed to test this possibility.

Additionally, poverty has been linked with poor sleep quality [19–21, 24, 59], and Hurley et al. demonstrated that living in a high deprivation index area was associated with reduced melatonin metabolite 6-sulfatoxymelatonin after adjusting for alcohol consumption, smoking, BMI, use of exogenous hormones, medications, coffee consumption, and physical activity [60]. Although urban light at night in populations with low socioeconomic status might contribute to melatonin suppression, one can’t exclude other possible environmental factors [60]. Another potential mediating factor between social disadvantage and melatonin suppression could be maternal mental health. Indeed, lower socioeconomic status is linked to risk for psychiatric disorders [74, 75]. Additionally, women with psychiatric disorders often have indicators of disruption in pregnancy, including lower nighttime melatonin concentrations, diurnal melatonin phase advance, and lower melatonin production in late pregnancy [76]. Melatonin disruption in pregnancy may also confer risk for worse postpartum mental health outcomes, as later sleep timing and a longer phase angle between melatonin onset and sleep timing in pregnancy predicts higher symptoms of postpartum mood and anxiety disorders [77]. Additional work is needed to examine the longitudinal associations between social disadvantage, mental health, and melatonin rhythms across the perinatal period. Moreover, sleep disruption is associated with negative reproductive outcomes [30–36], including altered development in neonates [78–80], but the mechanisms underlying this relationship are unknown. Future work should address whether weak melatonin rhythms contribute to poor reproductive outcomes among individuals with high social disadvantage.

### Strengths and limitations

We note three major strengths of our study. First, we measured diurnal melatonin profiles during pregnancy in a large, diverse cohort. Second, our saliva collection strategy caused minimal disruption to participants’ daily activities, which was likely important for recruiting and retaining participants who have traditionally been under-represented in studies. Pregnant people with high social disadvantage may have hurdles to participating in research studies, such as time away from work and family or limited transportation. Sample collection in real-life settings likely maximized participation. Third, we collected samples over a 24-hour period at three timepoints in pregnancy, permitting thorough characterization of diurnal melatonin cycles during pregnancy.

Our study also has several important limitations. First, only approximately one-third of participants supplied saliva samples for all three trimesters. We also observed that the lack of reporting annual income was more common among participants who did not contribute saliva samples. Second, although we asked participants to collect saliva at 1800 and every 4 hours thereafter for 24 hours, and all participants provided nighttime samples, ~10% of samples were collected over 30 minutes earlier or later than the requested time. We attempted to mitigate this limitation by using cosinor analysis to fit the data according to the actual collection times. Although cosinor fitting may not accurately model the sharp nocturnal rise and plateau of melatonin, cosinor-derived peaks and mesors strongly correlated with the raw values. Third, we did not collect data on sleep timing; thus, melatonin collection was timed by clock hour rather than relative to an indicator of the biological clock. Fourth, we did not control the participants’ environments, so participants may have been exposed to light at night when collecting saliva samples. However, our approach allowed us to measure diurnal melatonin concentrations in real-world settings rather than in a controlled laboratory environment. Fifth, we did not have information regarding whether the participants worked night or rotating shifts. Sixth, we excluded non-English speakers and pregnant teens. As these populations generally have higher SDS than English speakers and pregnant adults, their exclusion could affect generalizability. However, we speculate their inclusion would have strengthened our findings. Finally, we did not have complete medication data for all participants. Certain medications likely alter protein-binding of melatonin in the blood [81–84] or cross the blood-placenta barrier (e.g. betamethasone) [85] and thus affect salivary melatonin concentration, although plausibly less so than with plasma measurements [86].

## Conclusions

In conclusion, analysis of a large, diverse cohort of pregnant individuals shows that, on average, diurnal melatonin increases throughout pregnancy, but individuals with high social disadvantage have smaller diurnal melatonin increases throughout pregnancy than those with low social disadvantage. Social disadvantage, an amalgam of factors, could disrupt diurnal melatonin secretion. If future studies show that weak melatonin rhythms predict poor reproductive outcomes, then researchers should focus on identifying strategies to intervene – either via behavioral changes or pharmacologic therapy – to mitigate this risk.

## Condensation

Pregnancy-associated increase in salivary diurnal melatonin concentration mean and amplitude were blunted or even reversed in participants with greater social disadvantage.

## Supplementary Material

Supplemental_Data_file_zpaf064

## Data Availability

The data are not publicly available as the minimal data set for this study on pregnant participants contains identifying patient-level data that cannot be suitably de-identified or aggregated. Additionally, a subset of participants did not consent for future research in the informed consent form approved by the Institutional Review Board at Washington University in St. Louis. Data are available from the corresponding author upon reasonable request.
