# Supplementary material for: Social disadvantage is associated with impaired increase in salivary diurnal melatonin amplitude throughout pregnancy
Source: Sleep Adv. 2025 Sep 30;6(4):zpaf064. doi: 10.1093/sleepadvances/zpaf064 (PMC12535763; doi:10.1093/sleepadvances/zpaf064)
Supplement: Supplemental_Data_file_zpaf064 [file supplemental_data_file_zpaf064.pdf]

Title: Social disadvantage is associated with impaired increase in salivary melatonin amplitude throughout pregnancy.

Authors: Ronald MCCARTHY*[,1,2], Erin L. REINL*[,1,2], Patricia K. STRUTZI[3,], Peinan ZHAO[1], Andrea ESPARZA[1], Emma R. SASS[1], Meridith SCHOENING[1], Pranita KAGINELE[1], Naiqi SHI, Madeline CANAL, BA[1], Jessica E. CHUBIZ[1], Justin C. FAY[5#], Emily S. JUNGHEIM[1,6#], Nandini RAGHURAMAN[1], Lindsey N. KENT[1,2], Rebecca C. COX[7], PhD, Antonina I. FROLOVA[1,2], Erik D. HERZOG[8], and Sarah K. ENGLAND[1,2]

*These authors are co-first authors.

Affiliations:

[1] Department of Obstetrics and Gynecology, Washington University School of Medicine, St. Louis, MO, USA

[2] Center for Reproductive Health Sciences, Washington University School of Medicine, St. Louis, MO, USA

[3] Department of Anesthesia, Washington University School of Medicine, St. Louis, MO, USA

[4] Department of Genetics, Washington University School of Medicine, St. Louis, MO, USA

[5] Department of Biology, University of Rochester, Rochester, NY, USA

[6] Department of Obstetrics and Gynecology, Northwestern University Feinberg School of Medicine, Chicago, IL, USA

[7] Department of Psychological and Brain Sciences, Washington University in St. Louis, St. Louis, MO, USA

[8] Department of Biology, Washington University in St. Louis, St. Louis, MO, USA

[#] denotes present academic affiliation

Corresponding Author: Sarah K. England, PhD., 425 S. Euclid, Campus Box 8064, St. Louis, MO 63110, 314-286-1798, englands@wustl.edu

Funding: This work was supported by the National Institutes of Health grant R01 HD037831 (to SKE), the March of Dimes (to SKE, EDH, ESJ, and JF), and the Washington University Department of Obstetrics and Gynecology.

Title: Social disadvantage is associated with impaired increase in salivary diurnal melatonin amplitude throughout pregnancy.

Condensation: Pregnancy-associated increase in salivary diurnal melatonin concentration mean and amplitude were blunted or even reversed in participants with greater social disadvantage.

**Supplemental Table 1: Demographics of study cohort. Participants were stratified by completion of all 3 sample collections.**				
	**Total Cohort (n=921)**	**Incomplete samples (n=635)**	**Complete 3 samples (n=286)**	***P* value**
Age (years) at enrollment	28.25±5.43	27.66±5.55	29.55±4.92	< 0.001
BMI (kg/m^2) at first visit	29.06±8.24	29.48±8.38	28.14±7.87	0.02
Obese (%, BMI ≥ 30 kg/m^2)	340 (36.9)	252 (39.7)	88 (30.8)	0.01
Smoker (%)	93 (10.1)	80 (12.7)	13 (4.5)	< 0.001
Nulliparous (%)	379 (41.2)	234 (36.9)	145 (50.7)	< 0.001
Race (%)				< 0.001
Black or African American	469 (50.9)	374 (58.9)	95 (33.2)	
White	412 (44.7)	234 (36.9)	178 (62.2)	
Asian	18 (2.0)	12 (1.9)	6 (2.1)	
American Indian or Alaskan Native	3 (0.3)	1 (0.2)	2 (0.7)	
Unknown or Other	19 (2.0)	14 (2.2)	5 (1.7)	
Employment (%)				< 0.001
Yes	667 (72.4)	437 (68.8)	230 (80.4)	
No	185 (20.1)	144 (22.7)	41 (14.3)	
Student	28 (3.0)	19 (3.0)	9 (3.1)	
Unknown	41 (4.5)	35 (5.5)	6 (2.1)	
Income to need ratio	4.62±5.93	3.83±5.36	6.34±6.73	< 0.001
Hypertension (%)	96 (10.4)	70 (11.1)	26 (9.1)	0.43
Pregestational Diabetes (%)	23 (2.5)	15 (2.4)	8 (2.8)	0.89

Values are mean ± SD or N (%).
SDS, social disadvantage score; BMI, body mass index.
Continuous variables are presented as mean±SD and compared using Student's *t*-test. Categorical variables are presented as n (%) and compared using χ^2 test.

**Supplemental Table 2: Medians and IQR of saliva sample collection time difference to scheduled time. Values are shown in minutes.**							
	**Sample 1**	**Sample 2**	**Sample 3**	**Sample 4**	**Sample 5**	**Sample 6**	**Sample 7**
**Time**	**1800**	**2200**	**0200**	**0600**	**1000**	**1400**	**1800**
**Trimester 1**	0 (0, 7.0)	0 (0, 6.0)	0 (0, 4.0)	0 (0, 7.0)	0 (0, 10.0)	0 (0, 11.0)	0 (0, 8.0)
**Trimester 2**	0 (0, 7.0)	0 (0, 5.0)	0 (0, 3.0)	0 (0, 3.5)	0 (0, 10.0)	0 (0, 10.0)	0 (0, 10.0)
**Trimester 3**	0 (0, 2.5)	0 (0, 2.0)	0 (0, 1.0)	0 (0, 1.0)	0 (0, 3.0)	0 (0, 4.0)	0 (0, 5.0)

Time differences are shown in minutes as median (Q1, Q3)

Supplemental Table 3: Percentage of samples collected within 30 minutes of requested time.							
	**Sample 1**	**Sample 2**	**Sample 3**	**Sample 4**	**Sample 5**	**Sample 6**	**Sample 7**
**Time**	**1800**	**2200**	**0200**	**0600**	**1000**	**1400**	**1800**
**Trimester 1**	91.9%	89.2%	90.4%	86.5%	87.1%	87.7%	89.8%
**Trimester 2**	89.3%	88.7%	89.7%	87.5%	87.8%	85.2%	89.1%
**Trimester 3**	90.5%	91.7%	89.9%	88.1%	88.9%	86.9%	91.9%

Supplemental Table 4: Rate of change for diurnal melatonin profile parameters according to the linear mixed model with sensitivity analysis (patients with all 3 samples).			
**Melatonin Parameter**	**Baseline (pg/ml)**	**Slope (pg/ml per week)**	***P value***
Amplitude	10.4±0.6	0.06±0.02	<0.001
Mesor	10.1±0.5	0.17±0.02	<0.001
Mean	9.3±0.4	0.16±0.02	<0.001
Maximum	25.1±1.1	0.20±0.04	<0.001

Values are estimated coefficients from linear mixed-effects models. P value tests significant slope.

Supplemental Table 5: Comparison of diurnal melatonin profile parameters across trimesters with sensitivity analysis (only include samples collected within 30 mins of requested time).					
		**Fitted**		**Raw data**	
Trimester	N	Amplitude (pg/ml) (CI)	Mesor (pg/ml) (CI)	Maximum (pg/ml) (CI)	Mean (pg/ml) (CI)
1	323	9.7 (5.3, 15)	13.1 (8.9, 17.2)	27.2 (17.6, 37.5)	12.6 (8.5, 16.2)
2	582	9.0 (4.0, 15)	14.2 (9.5, 19.5)	29.3 (18.4, 41.9)	13.8 (9.5, 18.7)
3	597	9.0 (3.3, 17.1)	16.4 (10.8, 23.0)	32.5 (18.7, 45.5)	15.7 (10.5, 21.8)

Values are estimated coefficients from linear mixed-effects models, shown as median (Q1, Q3)

Supplemental Table 6: Rate of change for diurnal melatonin profile parameters according to the linear mixed model with sensitivity analysis (only include samples collected within 30 mins of requested time).			
**Melatonin Parameter**	**Baseline (pg/ml)**	**Slope (pg/ml per week)**	***P value***
Amplitude	9.7±0.5	0.05±0.02	0.019
Mesor	11.9±0.5	0.18±0.02	<0.001
Mean	11.4±0.4	0.14±0.02	<0.001
Maximum	27.0±1.0	0.15±0.04	<0.001

Values are estimated coefficients from linear mixed-effects models. P value tests significant slope.

**Supplemental Table 7. Association between SDS and diurnal melatonin profile parameters by trimester with sensitivity analysis (only include samples within 30 mins to the instructed time).**			
	Low SDS (< 0)	High SDS (>0)	*P* value
**Trimester 1**			
Amplitude (pg/ml)	11.7 (7.6, 16.5)	6.4 (3.1, 11.9)	< 0.001
Mesor (pg/ml)	13.4 (10.4, 17.3)	12.6 (7.8, 17.2)	0.06
Maximum (pg/ml)	30.4 (22.7, 38.7)	22.6 (14.6, 35.3)	< 0.001
Mean (pg/ml)	12.7 (9.6, 15.9)	11.7 (7.7, 16.2)	<0.001
**Trimester 2**			
Amplitude (pg/ml)	11.9 (7.5, 17.9)	4.5 (2.3, 10)	< 0.001
Mesor (pg/ml)	15.6 (11.6, 20.5)	11.5 (7.7, 17.8)	< 0.001
Maximum (pg/ml)	34.2 (22.5, 43.3)	22 (12.8, 34)	< 0.001
Mean (pg/ml)	14.7 (10.7, 18.7)	11.1 (7.4, 17)	< 0.001
**Trimester 3**			
Amplitude (pg/ml)	14.3 (9, 19.5)	4.0 (1.9, 8.3)	< 0.001
Mesor (pg/ml)	18.1 (13.8, 23.5)	13.1 (8.2, 21.5)	< 0.001
Maximum (pg/ml)	37.4 (26.5, 46.3)	23.2 (14.1, 36.3)	< 0.001
Mean (pg/ml)	16.5 (12.8, 21.4)	12.6 (8.2, 20)	0.001

SDS shown in (median (IQR)). Group comparison was conducted using Wilcoxon rank-sum test.

**Supplemental Table 8: Rate of change for diurnal melatonin profile parameters according to the linear mixed model with sensitivity analysis (patients with significant cosinor fits).**			
**Melatonin Parameter**	**Baseline (pg/ml)**	**Slope (pg/ml per week)**	***P value***
Amplitude	10.7±0.5	0.10±0.02	<0.001
Mesor	10.1±0.4	0.26±0.02	<0.001

Values are estimated coefficients from linear mixed-effects models. P value tests significant slope.

Supplemental Figure 1. The histogram of SDS shows bimodal pattern.

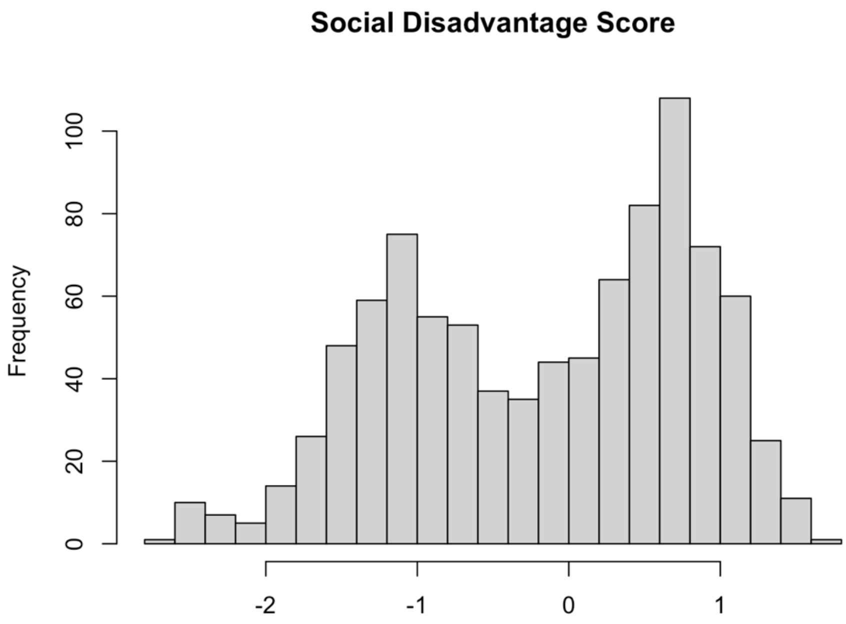

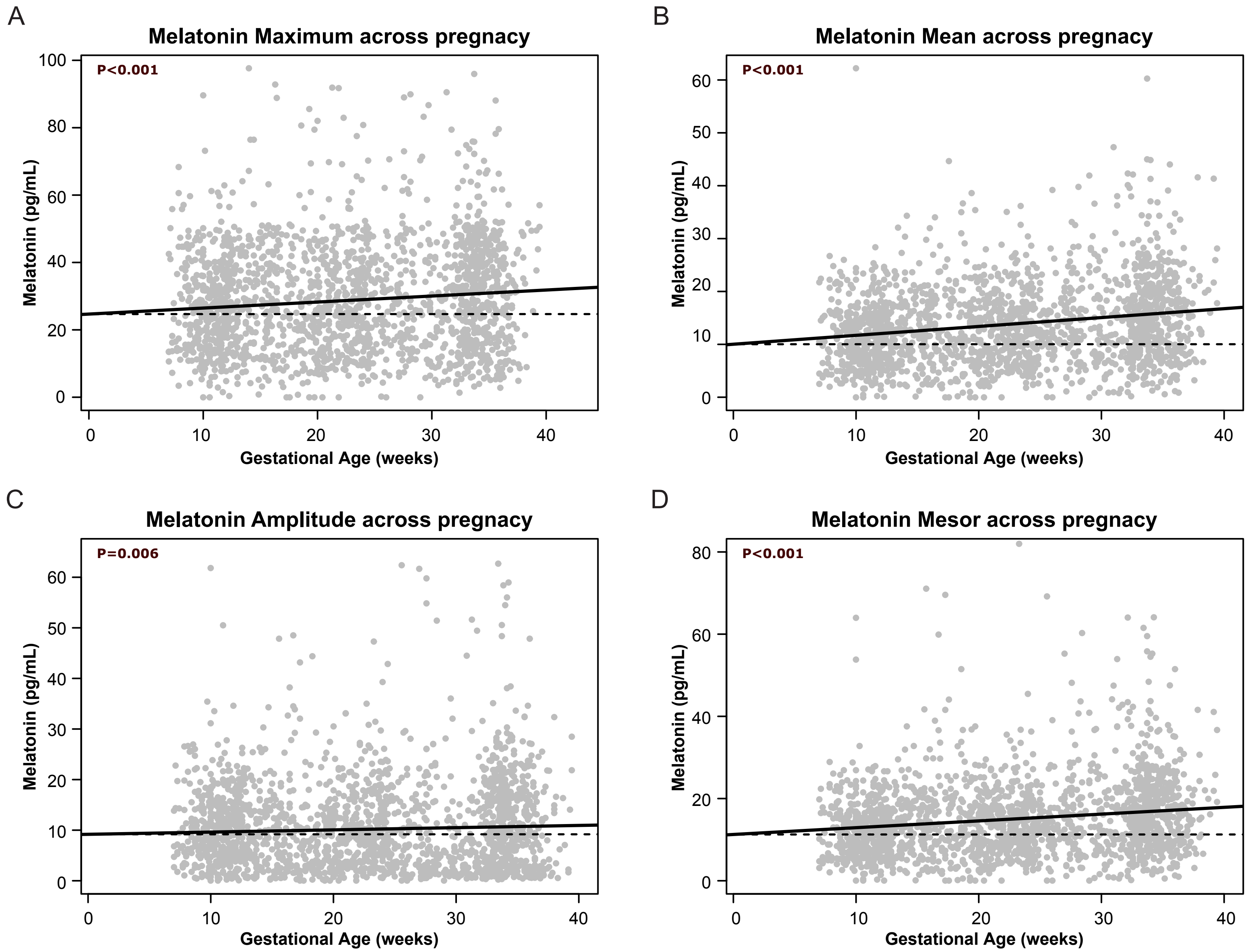

Supplemental Figure 2. Daily melatonin rhythms increase during pregnancy. Melatonin concentration (A) maximum, (B) mean, (C) amplitude, and (D) mesor increased from baseline through the third trimester. Points represent values from individual women. Solid lines represent average increase over time according to the linear mixed model. Dashed lines represent the calculated baseline concentration at the beginning of pregnancy (the intercept of the linear mixed model).
